# 

**Published:** 1913-07

**Authors:** 


### BUFFALO MEDICAL JOURNAL

### INDEX FOR ANNUAL VOL. 68

**From August, 1912, to July, 1913**

### Contributors of Original Articles.

Bassler, Anthony, New York, Medical versus Surgical Means of Diagnosis and Treatment of Gastro-intestinal Diseases, 135, Oct.

Belcher, Wm. W., Rochester, Factors Entering Into the Maintenance and Control of Free Dental Dispensaries, 382, Feb.

Bishop, Louis Faugeres, New York, The Pulse and Its Observation by Nurses, 197, Nov.

Bonney, Charles W., Philadelphia, Mammary Cancer, 71, Sept.

Brady, William, Elmira, Dens Sana in Corpore Sano, 389, Feb.

Crothers, T. D., Hartford, Home and Office Treatment of Inebriety, 253, Dec.

Doern, W. G., Milwaukee, Pituitary Extract, 78, Sept.

Dowd, J. Henry, Buffalo, The Sins of the Father, 692, July.

Ewald, C. A., Berlin, Parenchymatous Gastric Hemorrhages, [illegible]59, May.

Foy, George, Dublin, Caesarian Section, 8, Aug.

Hayd, Herman E., Buffalo. The Necessity for Accurate Pre-operative Diagnosis and the Methods to be Employed in Intra-abdominal Lesions, 681, July.

Hektoen, Ludvig, Chicago, Immunity, 63 Sept., 127 Oct., 190 Nov.

Hennington, C. W., Rochester, Tubal Pregnancy Within Wall of Uterus, 144, Oct.

Hennington, C. W., Rochester, Dr. E. S. Munn, the Rochester Opthalmologist, 264, Dec.

Hesse, Berlin, Dr. Freidmann's Curative and Prophylactic Vaccine, 499, April.

Hodgetts, Charles A., Ottawa, Pollution of Lakes and Rivers from the Canadian Standpoint, 307, Jan.

Keyes, Regina Flood, Buffalo, Notes of Trip Around the World, 586, May.

Knoll, John G. W., Buffalo, Early Diagnosis in Ectopic Gestation, 269, Dec.

Le Breton, Prescott, Buffalo, Some Practical Points Concerning the Operative Treatment of Bow Leg and Knock Knee, 503, April.

Le Breton, Prescott, Buffalo, Simple Portable Apparatus for Application of Plaster Jackets in Hyperextension, 626, June.

Lewis, F. Parke, Buffalo, Is Optometry the Practice of Medicine, 319, Jan.

Lewis, Joseph S., Buffalo, Abnormalities of Appendix Due to Disease, 251, Dec.

McKernon, James F., New York, Practical Points in the Diagnosis and Treatment of Acute and Chronic Aural Suppuration and Sequellae, 459 March, 513 April.

Park, Roswell, Buffalo, Fracture of Atlas, Separation of Fragment and Its Subsequent Extrusion through the Mouth, the Unique Case of Dr. James P. White, 312 Jan.

Park, Roswell, Buffalo, Report of 14 Cases of Spina Bifida and 1 of Sacrococcygeal Tumor, 437, March.

Potter, William Warren, late of Buffalo, Three Years with the Army of the Potomac, 13, Aug., 81, Sept., 148, Oct.

Prentice, Charles F., New York, Address before Optical Society of the State of New York, 314, Jan.

Quinton, W. W., Buffalo, Neosalvarsan, 80, Sept.

Quinton, W. W., Buffalo, Outline for Treatment of Eczema, 269, Dec.

Quinton, W. W., Buffalo, Much Neglected Branch of Dermatology, 567, May.

Reed, Boardman, Alhambra, Cal., Examinations of the Digestive Organs, Chronic Disease Generally, 621, June.

Richter, Julius, Buffalo, Simplified Technique in House Operations, 572, May.

Schreiner, Bernard F., Buffalo, Primary Carcinoma of Nipple, 572, May.
Sharp, Edward Affleck, Buffalo, Symptomatic Epilepsy, 580, May.
Shurley, Burt L., Detroit, Early Diagnosis of Pulmonary Tuberculosis, 398, Feb.
Smith, Edward T., Buffalo, Organotherapy, 20, Aug.
Smith, F. C., Keokuk, Ill., Clinical Report of Cases Treated with Phyacogen, 392, Feb.

Spangenthal, Joseph, Buffalo, The Mole, Relation to Malignant Disease of Skin, 146, Oct.
Stevens, Rollin H., Detroit, Isotonic Sea Water in Therapeutics, 369, Feb.

Trick, Harry R., Buffalo, Psychologic Side of Anaesthesia, 447, March.

Van Allen, John W., Buffalo, Investments for Professional Men, 564, May.

Wright, Thew, Buffalo, Compound Fractures, 1, Aug.

### General Index

Abnormalities of Appendix Due to Disease, Joseph S. Lewis, 251, Dec.
Abortion, Early Crusade Against Criminal, Edit., 51, Aug.
Address before Optical Society of State of New York, Charles F. Prentice, 314, Jan.
Alcoholic Beverages, Edit., 278, Dec.
Alumni Association, Medical Department, University of Buffalo, Proceedings of 1913 meeting, 695, July.
American College of Surgeons, 644, June.
A. M. A., Proposed Extension of Membership, 525, April.
Anaesthesia, Psychologic Side of, Harry R. Trick, 447, March.
Are Germ Diseases Practically Noncommunicable, Edit., 524, April.
Atlas, Fracture of; Separation and Extrusion of Fragment, Roswell Park, 312, Jan.
Aural Suppuration, Practical Points in the Diagnosis and Treatment of Acute and Chronic, James P. McKernon, 452, March, 513, April.

Bow Leg and Knock Knee, Operative Treatment, Prescott Le Breton, 503, April.
Boy, Your, Edit., 276, Dec.
Buffalo General Hospital Bulletin, 168, Oct.
Buffalo, University of, Proceedings of 1913 meeting of Alumni Association of Medical Department, 695, July.

Caesarian Section, George Foy, 8, Aug.
Cancer, Mammary, Charles W. Bonney, 71, Sept.
Carcinoma, Primary of Nipple, Bernard F. Schreiner, [illegible]09, April.
Centenarians, 477, March, 640, June.
Christian Science, Edit., 417, Feb.
Clinical Report of Cases Treated with Phylacogen, F .C. Smith, 392 Feb.
Clinical Study of Lower Animals, Importance of, Edit., 175, Oct.
Cocaine in Diarrhoea, E. Fuld, 666, June.
Collegiate Year for Medical Matriculants, Edit., 707, July.
Collegiate Year, Significance of as a Requirement to Medical Matriculation, Edit., 415, Feb.
Compound Fractures, Thew Wright, 1, Aug.

Dens Sana in Corpore Sano, Wm. Brady, 389, Feb.
Dental Dispensaries, Factors in Maintenance and Control, W. W. Belcher, 382, Feb.
Dermatology, Much Neglected Branch of, W. W. Quinton, 567, May.
Digestive Organs, Chronic Disease Generally, Boardman Reed, 621, June.
Domestic Pet, Edit, 47, Aug.

Early Diagnosis of Pulmonary Tuberculosis, Burt L., 398, Feb.
Ectopic Pregnancy, Early Diagnosis of, John G. W. Knoll, 271, Dec.
Eczema, Outline for Treatment, W. W. Quinton, 269, Dec.
English Words, Source of, 528, April.
Epilepsy, Symptomatic, E. A. Sharp, 580, May.
Ethics and Professional Influence, Edit., 419, Feb.
Extension of Membership of. A. M. A., 525, April.

Fly Swatting, Edit., 634, June.
Fractures, Compound, Thew Wright, 1, Aug.

Fracture of Atlas, Separation of Fragment, Extrusion Through Mouth, Case of Dr. James P. White, Roswell Park, 312, Jan.
Friedmann Anti-tuberculosis Serum, Edit., 471, March.
Friedmann Curative and Prophylactic Vaccination, Dr. Hesse, 499, April.

Gastric Haemorrhage, Parenchymatous, C. A. Ewald, 559, May.
Gastro-intestinal Diseases, Medical vs. Surgical Treatment, Anthony Bassler, 524, April.
Germ Diseases, Are They Practically Non-communicable? Edit., 524, April.
Gestation, see Pregnancy.
Graduating Class, To the, Edit., 588, May.
Guarantee of Interneships, Edit., 521, April.

Haemorrhage, Parenchymatous Gastric, C. A. Ewald, 559, May.
Happy New Year, Edit., 335, Jan.
Home and Office Treatment of Inebriety, T. D. Crothers, 253, Dec.
Humidity, Edit., 228, Nov.

Immunity, Ludwig Hektoen, 63, Sept., 127, Oct., 190, Nov.
Imposing Front, Edit., 632, June.
Income Tax, Edit., 470, March.
Inebriety, Home and Office Treatment of, T. D. Crothers, 253, Dec.
Interneship, Guarantee of, Edit., [illegible]21, April.
Intra-Abdominal Lesions, Necessity for Accurate Preoperative Diagnosis and Methods to be Employed in, Herman E. Hayd, 681, July.
Investments for Professional Men, John W. Van Allen, 564, May.
Is Optometry the Practice of Medicine, F. Parke Lewis, 319, Jan.
Isotonic Sea Water in Therpeutics, R. H. Stevens, 369, Feb.

Kafir Orange, R. H. True, 665, June.
Keep Cool, Edit., 630, June.
Knock Knee and Bow Leg, Operative Treatment, Prescott Le Breton, 503, April.

Mammary Cancer, Charles W. Bonney, 71, Sept.
Medical Education, Economic Considerations, Edit., 640, June.
Medical vs. Surgical Means of Diagnosis and Treatment of Gastro-Intestinal Diseases, Anthony Bassler, 135, Oct.
Mole, Relation to Malignant Disease of Skin, Joseph Spangenthal, 146, Oct.
Mortality, Distribution of, Edit., 592, May.
Much Neglected Branch of Dermatology, W. W. Quinton, 567, May.
Munn, Dr. E. G., the Rochester Dermatologist, C. W. Hennington, 264, Dec.

Necessity for Accurate Pre-operative Diagnosis and the Methods to be Employed in Intra-abdominal Lesions, Herman E. Hayd, 681, July.
Neosalvarsan, W. W. Quinton, 80, Sept.
Nipple, Primary Carcinoma of, Bernard F. Schreiner, 509, April.
Number of Physicians in Europe, 532, April.
Number of Physicians in U. S., 360, Jan.

Operations, Simplified Technic for House, Julius Richter, 572, May.
Operative Treatment of Bow Leg and Knock Knee, Prescott Le Breton, 503, April.
Optical Society of State of New York, Address before, Chas. F. Prentice, 314, Jan.
Optometry, Is it the Practice of Medicine, F. Parke Lewis, 319, Jan.
Organotherapy, Edward X. T. Smith, 20, Aug.
Overcrowded Profession (1845) Edit., 232, Nov.

Parenchymatous Gastric Haemorrhage, C. A. Ewald, 559, May.
Past Year and Future, 46, Aug.
Phipps, Henry Psychiatric Clinic, A. W. Hurd, 637, June.
Phylacogen, Clinical Reports of Cases Treated with, F. C. Smith, 392, Feb
Physicians, Number in Europe, 532, April, in U. S., 360, Jan
Physicians, Statistics Regarding Schools of Practice, 715, July.
Phthisiophobia or Red Tape, Edit., 710, July.
Pituitary Extract, W. G. Doern, 78, Sept.
Plaster Jackets, Portable Apparatus, Prescott Le Breton, 626, June.
Platform, Our, Edit., 231, Nov.
Pollution of Lakes and Rivers From Canadian Standpoint, Chas. A. Hodgetts, 307, Jan.
Pollution of Water, Edit., 336, Jan.
Portable Apparatus for Application of Plaster Jackets in Hyperextension, Prescott Le Breton, 626, June.
Possibilities of Paternalism in Medicine, Edit., 106, Sept.
Pregnancy, Early Diagnosis in Ectopic, John G. W. Knoll, 271, Dec.

Pregnancy, Tubal Within Wall of Uterus, C. W. Hennington, 144, Oct.
Primary Carcinoma of Nipple, Bernard F. Schreiner, 509, April.
Progress in Public Utilities, 337, Jan.
Psychologic Side of Anaesthesia, Harry R. Trick, 447, March.
Pulse and its Observation by Nurses, Louis Faugeres Bishop, 202, Nov.

Quadruplets, 597 and 603, May.

Red Tape or Phthisiophobia, Edit., 710, July.

Sacro-coccygeal Tumor and Spina Bifida, Roswell Park, 437, March.
Sea Water, Isotonic, in Therapeutics, R. H. Stevens, 369, Feb.
Second Class Postal Rates, Edit., 51, Aug., 331, Nov.
Significance of the Collegiate Year as a Requirement to Medical Matriculation, Edit., 415, Feb.
Simplified Technic for House Operations, Julius Richter, 572, May.
Sins of the Father, J. Henry Dowd, 692, July.
Source of English Words, 528, April.
Spina Bifida and Sacro-coccygeal Tumor, Roswell Park, 437, March.
Suicide Statistics, Edit., 34, Aug.
Symptomatic Epilepsy, E. A. Sharp, 580, May.

Tax on Time of Medical Societies, Edit., 631, June.
Three Years With Army of the Potomac, W. W. Potter, 13, Aug., 81, Sept., 148, Oct., 197, Nov.
Toads and Warts, Edit., 636, June.
Touting for Quacks, Edit., 335, Jan.
Trip Around the World, Regina Flood Keyes, 586, May.
Tubal Pregnancy Within Wall of Uterus, C. W. Hennington, 144, Oct.
Tuberculosis, Early Diagnosis of Pulmonary, Burt L. Shurley, 398, Feb.
Tuberculosis, Friedmann's Curative and Prophylactic Vaccination, Dr. Hesse, 499, April.
Tuberculosis Hospitals, County, 597, May, 714, July.
Tuberculosis Hospitals, Three Kinds, Edit., 711, July.

Vivisection Legislation, Edit., 173, Oct.

Warts and Toads, Edit., 636, June.
Water Pollution, Edit., 336, Jan.
Water Pollution, see article by Chas. A. Hodgett's, 307, Jan.
Where Will the Empire State Stand? Edit., 50, Aug.
White Slave Workers, Warning to, Edit., 633, June.
Why do Quacks Advertise to Regular Physicians, Edit., 709, July.
Women in Medicine, Edit., 277, Dec.

OBITUARIES

Baker, Samuel B., 726, July.
Bellamy, Archibald, 541, April.
Belknap, Edward B., 725, July.
Belknap, Milton C., 725, July.
Bevier, Archibald M., 540, April.
Billings, John S., 540, April.
Black, Frances, 487, March.
Bowden, Margaret, 119, Sept.
Bostwick, Harris A., 358, Jan.
Bristow, Algernon T., 614, May.
Brown, Henry W., 540, April.
Brower, Charles S., 539, April.
Brooks, Jean E., 435, Feb.
Bunn, Albert C., 436, Feb.
Burchard, John W., 234, Nov.
Bussman, Paul F., 358, Jan.

Clark, Coleman N., 487, March.
Clift, Frederic, 613, May.
Cheesman, Bryon C., 663, June.
Cochran, Frank T., 120, Sept.
Collier, Albert V. D., 539, April.
Constantin, Jules, 360, Jan.
Curtin, Roland G., 612, May.

Darlington, Stanley H., 180, Oct.
Douglass, Adelbert J., 43[illegible], Feb.
Dolph, B. V., 612, May.
Duhring, Louis A., 662, June.

Eastman, Robert W., 726, July.
Ebstein, Wilhelm, 358, Jan.
Egan, James A., 613, May.
Evans, Albert J., 540, April.

Findlay, Francis, 358, Jan.
Finnegan, Christopher, 613, May.
Fitsgibbons, William E., 234, Nov.

Gillette, Myra A., 613, May.
Gorse, Charles A., 235, Nov.
Gunn, Robert A., 360, Jan.

Haight, Alfred W., 120, Sept.
Hailes, William, 58, Aug.
Hale, Colewell D., 487, March.
Harvey, Leon F., 359, Jan.
Hazeltine, Lewis H., 612, May.
Henchell, Alfred W., 59, Aug.
Horwitz, Orville, 613, May.
Hoyer, Frederick F., 120, Sept.
Huggins, William Q., 235, Nov.

Ivey, Ellis V., 59, Aug.

James, Theron, 663, June.
Jones, Frank A., 541, April.
Jones, Henry W., 487, March.

Keiser, Max A., 359, Jan.
Kiepe, Edward J., 359, Jan.
Kimball, Pardon L., 180, Oct.
Kitchen, William W., 294, Dec.
Knapp, Theodore P., 487, March.

Lake, Daniel E., 613, May.
L'Hommedieu, John B., 487, March.
Loughhead, William H., 235, Nov.

MacLeod, Norman K., 612, May.
McCartney, Mary E., 615, May.
McClellan, George, 612, May.
McClintic, T. B., 119, Sept.
McGee, W. J., 181, Oct.
McNaughton, Malcolm N., 59, Aug.
McPherson, John D., 294, Dec.
Maguire, William G., 435, Feb.
Mason, Daniel G., 121, Sept.
Manchester, Delos B., 120, Sept.
Mead, Viola, 294, Dec.
Miller, Roger W., 487, March.
Monroe, S. Jean S., 663, June.
Morrow, Prince A., 613, May.
Murray, D. M., 60, Aug.

O'Reilly, Robert M., 293, Dec.
Oldberg, Oscar, 539, April.
Osborne, C. S., 358, Jan.

Palmer, Lewis R., 234, Nov.
Pellette, Arthur H., 60, Aug.
Pfandhoefer, Louis B., 487, March.
Pierce, William B., 662, June.
Pitner, Franklin R., 293, Dec.
Post, Frank R., 435, Feb.
Potter, Zira H., 615, May.
Prosser, Wellington, 180, Oct.
Prudden, William H., 119, Sept.
Puron, Juan G., 59, Aug.

Reilly, Edmund A., 662, June.
Richards, Charles, 180, Oct.
Richards, John, 726, July.
Richardson, Maurice H., 119, Sept.
Rouse, Morris D., 612, May.
Runnion, Emma A. B., 663, June.

Segond, Paul, 294, Dec.
Selover, John R., 541, April.
Singer, Herman B., 235, Nov.
Sheldon, Herbert P., 235, Nov.
Shurley, E. L., 660, June.
Smith, Ernest B., 435, Feb.
Steele, Minot H., 539, April.
Strange, Joseph N., 180, Oct.
Strong, Sylvester E., 613, May.
Sutton, Orlando, 662, June.

Taylor, George W., 487, March.
Taylor, John J., 118, Sept.
Tripp, Allen G., 59, Aug.

Van Gieson, Ira, 614, May.

Walker, Hiram D., 540, April.
Wales, Zippie B., 58, Aug.
Warren, Earl P., 180, Oct.
Watson, William H., 435, Feb.
Wile, William C., 539, April.
Willett, W. L., 436, Feb.
Witter, George H., 540, April.
Winnie, Ephraim, 541, April.

BOOK REVIEWS

Abt, Isaac, Practical Medicine Series, 363, Jan., Textbook of Physiology, 617.
Adams, P. H., Pathology of the Eye, 185, October.
Am. Association of Genitro-Urinary Surgeons, Transactions of, 548, April.
Am. Larynological Rhinological and Otological Society, Transactions of, 545, April.
Am. Telephone & Telegraph Co., Annual Report of, 667, June.
Appleman, L. F., Progressive Medicine. 363, Jan.
Appleton, Leighton F., Progressive Medicine, 248, Nov.
Ashton, W. Easterly, Text Book on the Practice of Gynecology, 304, Dec.
Aulde, John, Chemic Problem in Nutrition, 364, Jan.

Baker, LaReine H., Race Improvement or Eugenics, 367, Jan.
Bassoe, Peter. Practical Medicine Series, 493, March.
Baun, William L., Practical Medicine Series, 496, March.
Bergey, D. H., Principles of Hygiene, 304, Dec.
Bernays, Thekla. Augustus Charles Bernays, a Memoir, 61, Aug.
Bernays, Augustus C., Golden Rules of Surgery, 492, March.
Bidwell. Leonard A., Minor Surgery, 493, March.
Blair, Vilray P., Surgery of Diseases of the Mouth and Jaws, 302, Dec.
Blechmann, Germain, Les Epanchments DuPericarde, 670, June.
Brill, A. A., Psychanalysis, 549, April.
Bonadaile, L. A., Manual of Elementary Zoology, 493, March.
Brooks, Harlow, Diseases of the Ceni-to-Urinary Organs and Kidney, 124. Sept.

Brockbank, E. M., Children, Their Care and Management, 186, Oct.
Brubaker, Albert P., Text Book of Human Physiology, 243, Nov.
Buffalo State Hospital, 42d Annual Report, 543, April.
Bulkley, L. Duncan, Diet and Hygiene in Diseases of the Skin, 498, March.
Bythell, W. J. S., and Barclay. A. E., X-ray Diagnosis and Treatment, 185, Oct.

Cabot, Richard C., Physical Diagnosis, 368, Jan.
Canal Zone Medical Association, Proceedings of, 545, April.
Carling, E. Rock, Treatment after Operation, 496, March.
Catalogue of Medical and Surgical Works Published in the U. S., 240, Oct.
Century of Population Growth in U. S., 365. Jan.
Chetwood, Charles H., The Practice of Urology, 492, March.
Clark, J. Bayard, Essays on Genito-Urinary Subjects 62, Aug.
Collected Papers by the Staff of St. Mary's Hospital, 184, Oct.
Complete Catalogue of Medical Publications, 495, March.
Cowing, W. H., Blood Pressure, 498, March.
Crandon, L. R. G., Surgical After-Treatment. 183, Oct.
Cropper, J. W., Further Researches into Induced Cell Re-Production and Cancer, 244, Nov.
Dayton, Hughes, Practice of Medicine, 241, Nov.
Dentists' Dairy, 546, April.
DeLee, Joseph B., Principles and Practice of Obstetrics, 550, April.
DeLorne, M. F., Manual of Pharmacy for Physicians, 249, Nov.
DeWitt. Katherine, Private Duty Nursing, 670, June.
Diagnostic Points in Gastro-Intestinal Disease, 498, March.
Differential Diagnosis, 498, March.
Durning-Lawrence, Sir Edwin, The Shakespeare Myth, 187, Oct.

Ehrenfried, Albert, Surgical After-Treatment, 183, Oct.
Emery, W. D'Este, Clinical Bacteriology and haematology for Practitioners, 248, Nov.
Evans. Willmot, Diseases of the Skin, 494, March.

Flint, Austin, Manuel of Ausculation and Percussion, 304, Nov.
Fortescue-Brickdale, J. M., Text Book for Nurses Anatomy, Physiology, Surgery and Medicine, 60, Aug.
Fox, Herbert, Elementary, Bacteriological and Protozoology, 242, Nov.
Fowler, Russell S.. Operating Room and the Patient, 671, June.
Freeman, Leonard, Skin Grafting, 495, March.

Gardner, William S., a Text Book of Gynecology, 123, Sept.
Garrison, Fielding H., a New Work on the History of Medicine, 491, March.
Gastro - Enterological Association, Transactions of the 14th Annual Meeting, 670. June.
Greene, Robert H., Diseases of the Genito-Urinary Organs and the Kidney, 124, Sept.
Groves, E. W.. Text Book for Nurses Anatomy, Physiology, Surgery and Medicine, 60, Aug.
Grulee, Clifford G., Infant Feeding, 184, Oct.

Hare, Hobart A., Progressive Medicine. 248, Nov., 363, Jan.
Hare, Hobart A., Text Book of Practical Therapeutics, 303, Dec.
Harrison, L. W., Gonococcal Infections, 126, Sept.
Hart, Bernard, Psychology of Insanity, 364, Jan.
Head, Gustavus P., Practical Medicine Series, 302, Dec., 548, April.
Heisler, John C., Practical Anatomy, 249, Nov.
Herringham, W. P., Kidney Diseases, 125, Sept.
Hirst, Barton C., Text Book of Obstetrics, 305, Dec.
Hirschman, Louis J., Handbook of Diseases of the Rectum, 494, March.
Hooker, Albert H., Chloride of Lime in Sanitation, 544, April.
Hrdlicks, Ales, Bureau of Am. Ethnology, 244, Nov.
Hun, Henry, Atlas of Differential Diagnosis of Diseases of the Nervous System, 498, March.

Infant Mortality and Milk Stations, 62, Aug.

Jackson, George T., a Treatise on Diseases of the Hair, 245, Nov.
Jamieson, W. Allan, The Care of the Skin in Health, 185, Oct.

Keen, W. W., Keen's Surgery, 616, May.
Kemp, Robert C., Diseases of the Stomach, Intestines and Pancreas, 301, Dec.

Laboratory Data from the Department of Experimental Medicine, 492, Mar.
Lewers, Arthur N. H., Practical Text Book of the Diseases of Women, 243, Nov.
Lounsbery, Harriet C., Making Good on Private Duty, 240, Nov.
Lydston, G. Frank, The Blood of the Fathers, 241, Nov.
Lydston, G. Frank, a Privileged Medical Class, 669. June.

McFarland, Joseph, Text Book upon the Pathogenic Bacteria and Protozoa, 304, Dec.
McKisack, Henry L., Systematic Case-Taking, 667, June.
McLaren, Archibald, Transactions of the Am. Surgical Association, Vol. 13, 436, Feb.

Marchildon, John W., The Wassermann Reaction, 241. Nov.
Marshall, C. Devereux, Diseases of the Eye, 436, Feb.
Medical Directory of N. Y., N. J., and Conn., 250, Nov.
Medical Record Visiting List, 303, Dec.
Merck's Annual Report of Recent Advances in Pharmaceutical Chemistry and Therapeutics, 494, March.
Miles, Alexander, Manuel of Surgery, 186, Oct.
Mitchell. May, Practical Medicine Series, 363, Jan.
Mix, Charles L., Practical Medicine Series, 302, Dec., 548, April.
Morland, Egbert, Tuberculin Treatment, 126, Sept.
Morris, Roger S., Clinical Laboratory Methods, 670, June.
Mortality Statistics, 1909. 365, Jan.
Moynihan, B. G. A., Duodenal Ulcer, 124, Sept.
Muhlrad, Puccio y., Annuario Medico Sud Americano, 1912, 364, Jan.
Mulzer, Paul, Therapy of Syphilis, 367, Jan.
Murphy, John B., Surgical Clinics, 186. Oct., 301, Dec., 493, March, 669, June.
Murphy, John B., Contribution to the Surgery of Bones, Joints and Tendons, 546, April.
Murphy, J. Keogh, Practitioner's Encyclopedia of Medicine and Surgery, 246, Nov.
Murrill, William, What to do in Cases of Poisoning, 183, Oct.
Muscle Training in the Treatment of Infantile Paralysis, 547, April.

New Towns and Business Opportunities, 615, May.
N. Y. State Museum Bulletin, 244, Nov.
Noeberg, Geo. B., Golden Rules of Gynecology, 615, May.

Obstetric Charts in Colors, 122. Sept.

Park, Roswell, The Evil Eye, Thanthology and Other Essays, 495, Mar.
Peregrinus, Medicus, Men, Manners and Medicine, 548, April.
Pickerill, H. P., Stomatology in General Practice, 61, Aug.
Pilcher Hospital, Year Book, 300, Dec.
Physician's Visiting List, 303, Dec.
Polk's Medical Directory of North America. 1912, 360, Jan.
Pollock, C. E., Gonococcal Infections, 126, Sept.
Pottenger, Francis M., Tuberculin in Diagnosis and Treatment, 616, May.
Pottenger, Marion, Muscle Spasm and Degeneration in Intra-thoracic Inflammations, 248, Nov.
Practitioner's Visiting List, 303, Dec.
Progressive Medicine, 548, April.
Pusey, William A., The Care of the Skin and Hair, 61, Aug.

Radasch. Henry E., a Compend of Histology, 494, March.
Rassler, Anthony, Diseases of the Stomach and Upper Alimentary Tract, 545, April.
Rawling, L. Bathe, Landmarks and Surface Markings of the Human Body, 126, Sept.
Reilly, Thomas F., Building a Profitable Business, 363, Jan.
Report of the Commissioner of Education, 187, Oct.
Riviere, Clive, Tuberculin Treatment, 126, Sept.
Roberts. Stewart R., Pellagra, 125, Sept.
Robinson, Victor, An Essay on Hashesh, 122, Sept.
Roemer, Paul, Text Book of Ophthalmology, 436, Feb., 546, April.
Rockwood. Elbert W., Introduction to Chemical Analysis, 547, April.

Rose, A., Napoleon's Campaign in Russia, 548, April.
Ross, H. C. and E. H., Further Researches into Induced Cell-Reproduction and Cancer, 244, Nov.
Ross, F. W. Forbes, Cancer, the Problem of its Gensis and Treatment, 497, March.
Royal Academy of Medicine, Proceedings of 1913. 671, June.

Schottelius, Max, Bacteria, 367, Jan.
Schroeder, Henry H., Insurance Medicine, 671, June.
Scmitz, Henry, Pathology and Treatment of Diseases of Women. 247, Nov.
Scott, [illegible] J. E., Gould and Pyle's Cyclopedia [illegible]f Practical Medicine and Surgery, 123, Sept.
Scudder, Charles L., Tumors of the Jaws, 123, Sept.
Shaw, George Berbard, Doctor's Dilemma, 668, June.
Sight Saving as a National Movement, 667, June.
Simon, Charles E., An Introduction to the Study of Infection and Immunity, 302, Dec.
Simon, W.. Manuel of Chemistry, 245, Nov.
Sophian, Abraham, Epidemic Cerebro-Spinal Meningitis, 670, June.
Some Don'ts, Medical and Surgical, 497, March.
Starling, Ernest H., Principles of Human Physiology, 246, Nov.
State Board Exams, Questions and Answers, 300, Dec.
Stedman, Thomas L., a Reference Handbook of the Medical Sciences, 491, March.
Stevens. Thomas G., Diseases of Women, 549, April.
Stimson, Lewis A., Treatise on Fractures and Dislocations, 302, Dec.
Straus, Mrs. Lina G., Disease in Milk, the Remedy Pasteurization, 617, May.
Sutherland, G. A., Treatment of Disease in Children, 497, March, 548, April.
Sulzer's Short Speeches, 250, Nov.
Sulzer's Message of Public Health With Report of Special Public Health Commission, 547, April.
Stedman, Thomas L., Practical Medical Dictionary, 364, Jan.
Swedenborg, Emanuel, The Animal Kingdom, 184, Oct.

Thomson, Alexis, Manuel of Surgery, 186, Oct.
Thomson, H. Hyslop, Consumption in General Practice, 248, Nov.
Turner, William, Treatment after Operation, 498. March.
Tyrode, Maurice V., Pharmacology, 244, Nov.

U. S. Public Health Service, 1912, 666, June.

Vecki, Victor G., Sexual Impotence, 185, Oct.
von Noordan, Carl, New Aspects of Diabetes, 300, Dec.

Wallis, Sir Frederick, Surgery of the Rectum for Practitioners, 249, Nov.
Webster. Ralph W., Diagnostic Methods Chemical, Bacteriological and Microscopical, 60, Nov.
Whipham, T. Rowland C., The Medical Diseases of Children, 494, March.
Whiting, Frank V., Trespassers Killed on the Railroads, 182, Oct.
Wilcox, Reynold W., Materia Medica and Therapeutics, 242, Nov.
Williams, B. G. R. and E. G. C., Laboratory Methods, with Special Reference to the Needs of the General Practitioner, 123, Sept.
Wood. H. C., Jr., Pharmacology and Therapeutics, 247, Nov.
Woodwark, A. S., Manuel of Medicine, 304, Dec.

